# Burden of reduced FEV1 (<80% of predicted) and doctor diagnosed asthma and their association with smoking and BMI among urban adult population in Barrackpore, West Bengal (India)

**DOI:** 10.1186/1939-4551-8-S1-A65

**Published:** 2015-04-08

**Authors:** Kaushik Chakraborty, Ramandra Nath Mitra, Ramandra Nath Mitra, Peter L Thompson, AW Musk

**Affiliations:** 1 Barrackpore Population Health Research Foundation, India; 2 Sir Charles Gairdner Hospital, University of Western Australia, Australia; 3 School of Population Health (University of Western Australia), Australia

## Background

Bronchial asthma is a common and important cause of morbidity in adults. The World Health Organization (WHO) estimates that, the number of people suffering from asthma will exceed 100 million by 2025. Two recent national studies reported the burden of ‘self-reported’ and ‘diagnosed’ bronchial asthma to be 1.8% and 2.5% respectively in adult Indians. The diagnosis of Chronic Obstructive Pulmonary Disease (COPD) and bronchial asthma in developing countries are mostly empirical, and not dependent on ventilatory function evidence.

The study aims to measure the burden of reduced predicted forced expiratory volume in one second (FEV1), doctor diagnosed asthma, comparative relation of the two and to assess the role of smoking habits and body mass index (BMI) on both: reduced fev1and doctor diagnosed asthma.

## Methods

The cross sectional study was conducted among 3575 adult individuals. Subjects were recruited from longitudinal study of urban population cohort in Barrackpore, West Bengal. 'Asthma' was defined as a positive response to the question ‘Has been a doctor ever told you that you have asthma?’. Pulmonary function test was performed by trained and supervised spirometry technicians using computer based electronic spirometers. The predicted level of FEV1 was derived from Global Initiative for Asthma (GINA) guideline. The BMI was calculated by weight and height [weight (kg)/height (m)^2^] and classified according the WHO. The study was approved by the Institutional Ethics Committee of Barrackpore Population Health Research Foundation, India.

## Results

Overall 3.02% (108 individuals) of the population reported doctor diagnosed asthma, 3.02% male and 3.02% female. Reduced FEV1 (<80% of predicted) was reported in 11.94% (427) in the total study population, 15.45% (210) male and 9.79% (217) female. 319 (8.92%) of participants with FEV1<80% and 39 (1.37%) were FEV1<60% (not previously diagnosed).

39.22% of males and 0.22% of females were current smokers. Significant association was seen in the reduced FEV1 (<80% of predicted) in respect of never, former and current smoking (p<0.05) while asthma was not significantly associated with reduced FEV1 (p=0.25).

9.51% were found to be obese 4.49% (61 of 1359) male and 12.60% (279 of 2216) female. BMI was significantly associated with reduced FEV1 (p<0.05) but not with doctor diagnosed asthma.

## Conclusions

The prevalence of reduced FEV1 (<80% predicted) was high compared with doctor diagnosed asthma among the urban adults in the study area. Undetermined and undiagnosed asthma and or chronic obstructive pulmonary disease is an unrecognized significant non-communicable disease burden in this urban Indian community.

